# Lifestyle Intervention in Reducing Insulin Resistance and Preventing type 2 Diabetes in Asia Pacific Region: A Systematic Review and Meta-Analysis

**DOI:** 10.1007/s11892-024-01548-0

**Published:** 2024-07-31

**Authors:** Yingting Cao, Abha Shrestha, Amy Janiczak, Xia Li, Yang Lu, Tilahun Haregu

**Affiliations:** 1 School of Allied Health, Human Services and Sport, La Trobe University, Plenty Road, Kingsbury Dr, Bundoora, VIC 3086 Australia; 2 Non-communicable and implementation science lab, Baker Heart and Diabetes Institute, Alice Springs, Australia; 3 School of Psychology and Public Health, La Trobe University, Melbourne, Victoria, Australia; 4 Statistics Consultancy Platform, La Trobe University, Melbourne, Australia; 5 School of Sociology and Population Studies, Nanjing University of Posts and Telecommunications, Nanjing, China; 6 Melbourne School of Population and Global Health, The University of Melbourne, Melbourne, Australia

**Keywords:** Type 2 diabetes, Lifestyle intervention, Implementation, Asia pacific region

## Abstract

**Purpose of Review:**

To update the evidence of lifestyle interventions for the prevention of type 2 diabetes mellites (T2DM) in adults, particularly in the Asia Pacific region. The key questions to ask are: 1) How effective are lifestyle interventions in preventing T2DM among at-risk adults in the Asia Pacific Region? 2)What are the key characteristics of the implementation of lifestyle interventions for diabetes prevention?

**Recent Findings:**

Lifestyle interventions for the prevention of T2DM have been suggested to be effective. There is evidence of ethnic differences in some glycaemic and anthropometric outcomes.

**Summary:**

The meta-analysis suggested a significant result in reducing waist circumference (standardised mean difference − 019, 95%CI ( -0.31, -0.06)), and no significant effects in other outcomes. However, the implementation outcomes suggested lifestyle intervention might be a cost-effective and sustainable approach in T2DM particularly in countries in the Asia Pacific Region. The focus of lifestyle intervention in the Asia Pacific Region should not only lie in the effectiveness of the trial but a thorough evaluation of the implementation outcomes, as well as cultural adaptations, with the support of all stakeholders through all stages of the implementation.

**Supplementary Information:**

The online version contains supplementary material available at 10.1007/s11892-024-01548-0.

## Introduction

Type 2 Diabetes Mellitus (T2DM) undoubtedly has become a major public health concern both globally and regionally. The burden of T2DM is rising dramatically, with disproportionately higher prevalence and burden in lower-income countries [1]. In 2021, the International Diabetes Federation (IDF) estimated that 537 million people had diabetes worldwide, and this number is projected to reach 643 million by 2030, and 783 million by 2045 [2].

Three in four people with diabetes are living in low- and middle-income countries, with China, India, and Pakistan as the top three countries in the 10 leading countries with diabetes [3]. Not surprisingly, the leading regions of diabetes around the world in 2021 were the Western Pacific Region (WPR) (206 million) followed by Southeast Asia (90 million) [3]. This has been described as the rising global public health “tsunami” previously [4], and drastic steps and measures that need to be taken are urgently needed. There exist difficulties in comparison among studies in Asia and the Pacific, due to the differences in age groups, survey methodologies, and diagnostic criteria, as well as other aspects [4]. However, it is important to note that the majority of developing countries within the WPR and South Asia have shown escalating rates of diabetes prevalence [4]. In this review, we will include both WPR and Southeast Asia to call them the Asia Pacific Region according to the Federal Aviation Administration [5]. The focus of this review on the Asia Pacific region is due to the increased immigration (nearly one-third of the total Australian population was born overseas) to Australia from this region, including India, China, the Philippines, and New Zealand, which are ranked top 5 countries of birth, excluding Australia (with England as the top country of birth) [6].

Recent systematic reviews have explored lifestyle interventions and their impact on T2DM. A systematic review found lifestyle interventions significantly decreased the risk of T2DM compared to the control group in individuals with impaired glucose tolerance [48, 7]. Another recent review has explored ethnic differences in the effectiveness of lifestyle for T2DM suggesting lifestyle interventions similarly reduce T2DM incidence but can have different effectiveness on other glycaemic and anthropometric outcomes across ethnicities [7]. The review included more than half of the studies conducted in the United States and UK, and the dominant ethnic groups were European or Caucasian/white, although there are also other ethnic groups included, such as African Americans and Asians [7]. As mentioned above, countries in the Asia Pacific Region are currently experiencing a fast-increasing burden of T2DM, and an update of the literature on lifestyle intervention in this region is urgently needed to guide practice. In addition, given the difficulties of comparisons of lifestyle interventions for the prevention T2DM across countries, assessing the implementation outcomes is particularly important in settings with potential resource constraints. However, none of the systematic reviews mentioned above discussed the implementation of the lifestyle intervention in real world, but only focusing on effectiveness itself. Haw et al., [8] has confirmed the sustainability of lifestyle intervention to reduce the incidence of T2DM, however, interventions to preserve effects are needed. In another recent review on the feasibility of prevention for T2DM particularly in resource constrained countries, although confirming the potential evidence of lifestyle preventions for T2DM, implementation challenges exist from multiple layers (i.e., individual, societal and healthcare system [9].

Previous reviews have explored all literature up to 2020. Some studies in the aforementioned reviews [7, 10] even included studies published in more than 20 years ago. Therefore, it is necessary to review the literature published up to date with a focus on the Asia Pacific Region and discuss the current evidence gap. The aim of this systematic review is to discuss the impact of lifestyle interventions in prevention T2DM among people within the Asia pacific region. In addition, it aims to explore and discuss the implementation of lifestyle interventions in the Asia Pacific Region.

## Methods

### Data Sources and Search Strategies

A systematic literature search was conducted across multiple databases, including Medline, Embase, CINAHL, and Cochrane Central Register of Controlled Trials to identify relevant studies. All databases were searched between July and November 2023. The search strategies were developed via close consultation with an experienced medical librarian based on Medical Subject Headings (MeSH) terms with input from the authors. The initial search was developed in Medline, and the search strategies have been subsequently adapted to other databases criteria (i.e., Embase, CINAHL, and Cochrane Central Register of Controlled Trials). A hand search for additional articles was done in Google Scholar. The detailed search strategies and the use in different databases can be found in Supplemental material 1 and 2. In addition, we set the limits to all the search to “randomised controlled trials”, “published in the last 10 years” (i.e., 2013–2023), “full text” and “English language”. The updated version of Preferred Reporting Items for Systematic Reviews and Meta-Analyses” (PRISMA) [11] was referred to conduct the current systematic review and meta-analysis. The protocol was registered at PROSPERO (www.crd.york.ac.uk/PROSPERO) as CRD42023471474.

### Study Selection

Studies are included if (1) they included adults aged 18 years and older who are at risk of developing T2DM, including prediabetes, high BMI, history of gestational diabetes, family history of diabetes, elevated diabetes risk score, metabolic syndrome; (2) the participants live in the Asia Pacific Region: mainly includes countries in the Asia Pacific region according to the Federal Aviation Administration [5], as mentioned above (e.g., North East Asia, South Asia, South East Asia, and Pacific or West Pacific); (3) RCTs include any lifestyle interventions (e.g., dietary and/or physical activity) that aimed to prevent T2DM; (4) report at least one of the following outcomes as the study main outcome: T2DM incidence, fasting glucose, 2-h glucose (OGTT), HbA1C and/or implementation results. Studies are excluded if (1) they included participants aged < 18 years; (2) participants with diagnosed diabetes (type 1 or type 2); (3) included pregnant women; (4) Taking medication that would alter glycaemic outcomes; (5) Any interventions without diet or physical activity component (e.g., only supplement or pharmacological intervention); (6) combined lifestyles with drugs, supplements, or other treatment. In addition, all editorials, letters, commentaries, conference abstracts, theses/dissertations, study protocols, reviews and other reports are excluded.

Covidence [12] was used for titles and abstracts screen, as well as full texts review and data extraction. Title and abstract screening and full-text review were performed by TC and AS according to the eligibility defined above. Any discrepancy was checked and resolved by a third reviewer (TH). Data extractions were independently performed by YC, LY, AJ and AS, and any discrepancy were resolved by consensus via group discussion.

### Quality Assessments of the Studies

The JBI critical appraisal checklist for randomized control trials [13] was used to assess the quality of studies included in this review by two independent reviewers from the pool of reviewers (YC, LY, AJ and AS). There are 13 questions in total, assessing internal validity (bias related to selection and allocation, bias related to administration of intervention/exposure, bias related to assessment, detection, and measurement of the outcome, bias related to participant retention) and statistical conclusion validity. The risk of bias was assessed as either low, high, or unclear. All discrepancies and disagreements were resolved through consensus, or by a third reviewer that was not involved in the studies in the risk assessment stage.

### Data Extraction

Key relevant data from each of the selected studies were extracted using the template in Covidence, with modifications where needed. These data included study characteristics, including study title, first author, country of the study, publication year, sample size, intervention characteristics, follow-up length, and outcomes. Primary outcomes included T2DM incidence (the new cases reported at the end of the study point), glycaemic outcomes (fasting and 2-h glucose levels, and HbA1c). Secondary outcomes (if reported) included anthropometric measures (body weight, BMI, waist circumference), lifestyle behaviors (energy intake and physical activity) and implementation outcomes (cost-effectiveness, feasibility, sustainability, etc.)

### Data Synthesis and Analysis

The intervention effects were synthesized using random-effects meta-analysis models on fasting glucose, Hb1Ac, BMI, and waist circumference at 6 months follow-up, based on the available data for most studies. Standardized Mean Differences (SMDs) with 95% confidence intervals (CIs) were employed to represent continuous outcomes. The forest plots visually presented the effect sizes. Homogeneity among studies was assessed using the I² test, with I² > 50% indicating substantial heterogeneity. Following the Cochrane Handbook guidelines [14], an I² of 0–40% might not be important, 30–60% may represent moderate heterogeneity, 50–90% substantial heterogeneity, and 75–100% considerable heterogeneity. Publication bias was examined using funnel plots and Egger’s test. It’s important to note that Egger’s test may lack statistical power with a small number of studies. Therefore, according to Sterne et al., [15], funnel plot asymmetry tests were performed only when 10 or more studies were present. A two-sided P-value < 0.05 was considered statistically significant for all analyses. The meta package in R version 4.3.2 was utilized for conducting the analyses.

## Results

### Study Identification

The initial search of different databases identified 2046 studies. After removing duplicates, 1894 titles and abstracts were screened, and 67 studies were selected for full-text screening by removing studies for different reasons. The full-text studies were further reviewed and assessed for eligibility, and finally, 29 articles (25 original studies/trials) met inclusion criteria in this review. 13 studies were included in the meta-analysis with required data (Supplemental Fig. 1).

### Study Characteristics

Study characteristics and participants characteristics of the studies are presented in Table 1.

In total, all the 25 unique studies (28 articles/reports) included articles enrolled 27,167 participants across nine countries. More than half of the studies were conducted in China /Taiwan (*n* = 12) and India (*n* = 7), whereas the rest were studies from Thailand (*n* = 3), Australia (*n* = 1), Vietnam (*n* = 1), and Japan (*n* = 1). The characteristics of the studies included in this review are summarized in Table 1. Sixteen studies have a combination lifestyle intervention (1 used a telephone support program), mainly focused on both dietary and physical activity/exercise intervention, and 7 studies had physical activity/exercise intervention only and 3 studies had diet intervention only (1 through mobile app). Most studies included both genders, except one included man only with impaired glucose tolerance (IGT) [16] and four included women only with pre-diabetes [17, 18] or gestational diabetes [19, 20]. The interventions lasted from 3 to 12 months, with follow-up arranges from 1 to 30 years. All studies are conventional RCT study designs, except six studies [21–26] are clustered RCT.

### Risk of bias Assessment

The “Traffic Light” plot (Supplemental Fig. 2) and the “Rob Summary plot” (Supplemental Fig. 3) for quality assessment of the included studies are presented using the Robvis [27]. There are in total 13 domains, and 25 RCTs were assessed for the quality of the study. Please refer to the details on each of the domains in Supplemental Material 3.

### Meta-analysis and Publication bias

**Fig. 1:**
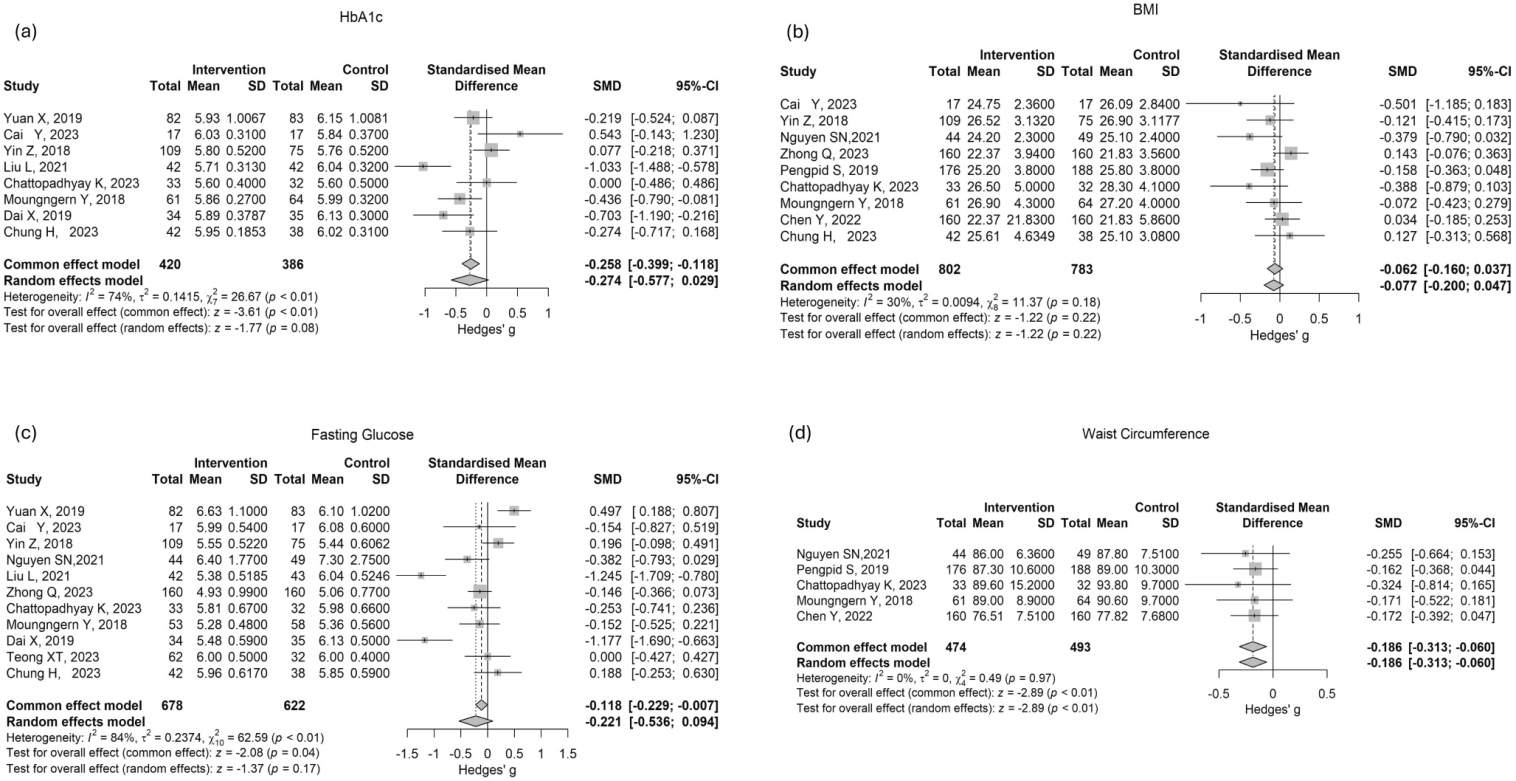
The effectiveness of lifestyle intervention on Hb1Ac, fasting glucose, BMI and waist circumference at 6-month

Meta-analysis was conducted for studies that reported results at 6 months for Hb1Ac, fasting glucose, BMI, and waist circumference wherever the data is available. The effect of lifestyle intervention on glycaemic outcomes seems to be not significant for HbA1c and fasting glucose (Fig. 1a). High heterogeneity (I^2^ = 74% and I^2^ = 84%) was seen for HbA1c and fasting glucose. For anthropometric outcomes, no effect has been shown in reducing BMI via lifestyle interventions (Fig. 1b) and a significant reduction has been shown in reducing waist circumference (standardised mean difference − 019, 95%CI ( -0.31, -0.06) ) (Fig. 1c). Whereas a moderate heterogeneity (I2 = 30%) was suggested for BMI, no heterogeneity (I2 = 0%) was found in waist circumference. Publication bias was only reported for the outcome included more than 10 studies (only for fasting glucose at 6 months) according to the guideline [14]. There was no asymmetric reported for fasting glucose at 6 months (Egger’s test *p* = 0.21).

**Table 1:** Characteristics of included studies

Author, year	Country	Study design	Endpoint (months)	Sample size	Intervention	Population	Outcome measures	Comments
Yin Z et al., 2018 [17]	China	RCT	12	184	Combined lifestyle intervention	Women with pre-diabetes, ages 25–65	Weight, fasting glucose, HbA1c and self-reported diet and physical activity	
Thankappan KR et al., 2018 [26] (Sathish T,2020 [28], Aziz Z 2018 [29])	India	Cluster RCT	24	1007	Combined lifestyle intervention	those with an Indian Diabetes Risk Score (IDRS) ≥ 60, 30–60 years	Fasting glucose, 2-hr glucose, HbA1c, body weight, BMI, waist circumference, incidence of T2DM, cost effectiveness	Same study, two additional implementation reports
Nanditha A et al., 2018 [16]	India	RCT	60	517	Combined lifestyle intervention	Men aged 35–55 with impaired glucose tolerance (IGT)	Fasting glucose, 2-hr glucose, BMI, waist circumference, energy intake, incidence of T2DM, physical activities	
Pengpid S et al., 2018 [30]	Thailand	RCT	12	443	Combined lifestyle intervention	35–65 years, with prediabetes and/or prehypertension	Fasting glucose, blood pressure, lipid profiles, and anthropometric measures	
Moungngern Y et al., 2018 [31]	Thailand	RCT	6	125	Combined lifestyle intervention	Pre-diabetes subjects	Fasting glucose, HbA1c, body weight, BMI, waist circumstance	
Yan J et al., 2019 [32]	China	RCT	12	105	PA only	Participants with prediabetes	Fasting glucose, 2 h glucose, HbA1c, BMI	
Dai X et al., 2019 [33]	China	RCT	24	172	PA only	Adults aged 55 to 75 years with a diagnosis of prediabetes	Fasting glucose, 2 h glucose, HbA1c, body weight, incidence of T2DM	
Aekplakorn W et al., 2019 [21]	Thailand	Cluster RCT	24	1903	Combined lifestyle intervention	Individual aged 30 to 65 years with impaired oral glucose tolerance	Fasting glucose, Body weight, BMI, lipid profile Incidence of T2DM	changes at 2-year follow-up
Yuan X et al., 2019 [34]	China	RCT	6	248	PA only	Individual with prediabetes	Fasting glucose, HbA1c, BMI, body weight, waist circumference, lipid profiles	
Liu L et al., 2021 [35]	China	RCT	12	128	PA only	Individuals with isolated impaired glucose	Fasting glucose, 2 h glucose, HbA1c	
Chen X et al., 2020 [22]	China	Cluster RCT	3	138	Diet only (via mobile app)	Adults with Prediabetes	Fasting glucose, HbA1c, body weight, BMI, lipid profiles, exercise activities, knowledge, dietary balance	
Gong Q et al., 2021 [36]	China	30-year follow-up of RCT	6,10,20,30 years	576	Combined lifestyle intervention	adults with impaired glucose tolerance	fasting glucose, incidence of T2DM, efficacy	Da Qing 30 year
Kaur H et al., 2021 [37]	India	RCT	3	120	Combined lifestyle intervention	prediabetic females	fasting glucose, 2 h glucose, HbA1c, body weight, BMI, waist circumference, lipid profile	
Raghuram N et al., 2021 [24]	India	Cluster RCT	3	4450	PA only	Individuals (age, 20–70 years) with prediabetes	Diabetes risk	RRR report
Kaur N et al., 2021 [18]	India	RCT	3	184	PA only	People with Indian Diabetes Risk Scoring (IDRS) ≥ 60	fasting glucose, 2 h glucose, HbA1c, body weight, BMI, waist circumference	
Sakane N et al., 2021 [25]	Japan	Cluster RCT	12	2607	Combined lifestyle intervention	participants aged 20–65 years with impaired fasting glucose	stages of change toward healthy eating and active exercise, fasting plasma glucose	stage of change framework
Nguyen SN et al., 2021 [23]	Vietnam	Cluster RCT	6	93	Combined lifestyle intervention			
Guo H et al., 2022 [38]	China	RCT	2.5	90	Diet only	Obese/overweight adults with impaired glucose regulation	weight, fasting blood glucose	
Liu Y et al., 2022 [39]	China	RCT	12	2865	Combined lifestyle intervention	Those with a prediabetes diagnosis	fasting glucose, 2 h glucose, HbA1c	
Chung H et al., 2023 [40]	China Taiwan	RCT	3	121	Combined lifestyle intervention	People with prediabetes	fasting glucose, HbA1c, BMI, physical activities	
Tandon N et al., 2022 [19]	South Asia (India, Bangladesh, and Sri Lanka)	RCT	12	1601	Combined lifestyle intervention	women with recent t gestational diabetes GDM	fasting glucose, Body weight, waist circumference, systolic blood pressure, Incidence of T2DM, energy intake	
Teong XT et al., 2023 [41]	Australia	RCT	18	209	Diet only	Adults at increased risk of developing T2DM	fasting glucose, HbA1c, body weight, BMI, waist circumference	
Cai Y et al., 2023 [42]	China	RCT	6	34	PA only	people with pre-diabetes	body composition (body weight and body fat distribution), plasma glucose, lipid and the homeostatic model assessment of insulin resistance (HOMA-IR), inflammatory cytokines	
Zhong Q et al. 2023 [20] (Chen 2022 [43])*	China	RCT	18	320	Combined lifestyle intervention	Adult women with a history of gestational diabetes mellitus	T2DM risk score, fast glucose, 2 h glucose, weight-related, behavioral and psychological variables	Same study, with different follow-up data
Chattopadhyay K et al., 2023 [44]	India	RCT	6	81	PA only	Adults with a fasting blood glucose level of 100–125 mg/dL	Fasting glucose, HbA1c, Body weight, BMI, weight circumference, blood pressure, lipid profiles, physical activities	

*same RCT reported results on different endpoints

### Implementation Outcomes

Several studies reported and discussed the implementation outcomes. Aziz et al., [29] discussed the evaluation of implementation of the K-DPP study by Thankappan [26], indicating that the K-DPP program led by peers was feasible and acceptable in changing lifestyle behaviours in high-risk population of developing T2DM. Another cost-effectiveness evaluation of the K-DPP study concluded a total of US$291.5 per diabetes case prevented and US$50 and US$150 were gained per quality-adjusted life years (QALY) for both health system and societal perspectives. Gong et al. [36] reported the 30- year follow-up of the Da Qing Diabetes Prevention Outcome study initiated in 1986, suggesting sustainable and effective lifestyle intervention to reduce T2DM incidence in people with impaired glucose tolerance, regardless of baseline fasting glucose level. Tandon et al., [19] reported the results of a randomised implementation pragmatic trial on the effectiveness of lifestyle interventions among previously diagnosed with gestational diabetes during pregnancy. The study reported a reduced fidelity affected by slow initial recruitment and subsequently COVD-19. Similarly, Chattopadhyay et al. [44] also reported promising feasibility in their RCT of implementing a yoga program in preventing T2DM, although some challenges towards the end of the study due to the COVID-19 lockdown in India. In a Chinese translational study investigating the cultural adaptation of evidence-based lifestyle intervention (i.e., Pathway to Health (PATH) program) for diabetes prevention in women with prediabetes has confirmed the feasibility and acceptability in community health centres, although large RCTs is needed [17]. A series of strategies including setting recursion goals and providing environmental support was provided in the cluster RCT led by Zhong et al., [20] suggesting the efficacy of the intensive lifestyle modification program among rural women with a history of gestational diabetes.

## Discussion

Our study has managed to update the latest evidence of the effectiveness and implementation outcomes of lifestyle intervention in the prevention of T2DM, with a focus on the Asia Pacific Region, which has been shown to increase T2DM burdens. Although not all the studies with all the outcomes were synthesized in the meta-analysis, the results suggested a significant result in reducing waist circumference, with no significant effect in other outcomes.

Differently from the previous review that found significant improvement in T2DM incidence, glycaemic and anthropometric outcomes, as well as ethnic differences in some of the outcomes [7], we did not find any significant improvement in glycaemic and anthropometric outcomes, except waist circumference. However, they focused on quite a huge range of ethnicities globally, while ours only focused on the Asia Pacific Region. Most outcomes have indicated a huge heterogeneity from the studies included. There is also a huge range of intervention lengths from 1.5 to 72 months, which were analysed as a whole, but not by intervention length. So, it is unclear whether the intervention is effective in short term or long term, as sustainability is critical in the evaluation of lifestyle interventions in preventing T2DM.

There is a significant reduction in waist circumference by lifestyle intervention found in our meta-analysis, with no heterogeneity detected (Fig. 1d), although only from five studies whichever with the available data. The result aligns with the systematic review which investigated ethnic difference, with a significant reduction in waist circumference also found in East and Southeast Asians (the ethnicity of our five studies included in the meta-analysis for waist circumference). Interestingly, only one study had physical activity only as the intervention [44], whereas the rest were all had combined lifestyle interventions (i.e., diet and physical activity approaches) [23, 30, 31, 43]. Central obesity has been suggested to be the strongest risk factor for cardiometabolic diseases, including T2DM in the Asia Pacific populations [45]. This may be attributed to the cultural and geographical characteristics of the region. Evidence has shown the effectiveness of culturally tailored lifestyle interventions for managing T2DM among Black African populations in the US [46], which suggests more research is needed for more culturally tailored lifestyle interventions in other populations.

In addition, another point we would like to stress is that prevention T2DM is complicated and involves multi-layer and multi-stage effort [9], we should not only rely on the effectiveness or effect size from meta-analysis to evaluate whether an intervention works or not. Lifestyle intervention including dietary and physical activity approaches, can be conducted in a various way, which is hard to compare with a simple drug intervention even using an RCT study design. As we know even an effective intervention may not necessarily be evenly implemented in reality, which is so-called “one size doesn’t fit all” [47]. It really requires all stakeholders at all stages of the intervention process to overcome contextual barriers, particularly in countries with constrained resources.

The major strength of this review is that we used the results of meta-analysis to demonstrate the current evidence on the effectiveness of lifestyle intervention in the prevention T2DM, with a focus on the Asia Pacific Region. Yet, we have also reported the implementation outcomes, to complement the gaps of lifestyle intervention in real-world situations. Some limitations though needed to be claimed: (1) meta-analysis was only conducted for some but not all studies for some outcomes at 6-month, however, this is due to available data from the studies; (2) some results from meta-analysis had high heterogeneity, and this could be done further by conducting sensitivity analysis; (3) not all studies included in the meta-analysis reported implementation outcomes. Therefore, the results from meta-analysis in this review should be interpreted with caution given the limitations mentioned above.

Future research in lifestyle interventions to prevent type 2 diabetes in Asia-Pacific countries should prioritize addressing the implementation gaps identified in the current literature. Firstly, there is a critical need for studies that assess the cultural appropriateness and adaptation of lifestyle interventions across diverse populations in the region. Research should explore how cultural norms, dietary habits, and socioeconomic factors influence the adoption and sustainability of preventive measures. Programs should take into account the sustainability and scalability of the intervention from the very beginning of the design phase. Additionally, there is a scarcity of long-term follow-up studies that evaluate the effectiveness of interventions beyond immediate outcomes, such as sustained behavior change and reduction in diabetes incidence over the years. Future research could also focus on developing and testing innovative delivery models, including digital health platforms and community-based programs tailored to the unique infrastructural and healthcare access challenges in various Asia-Pacific settings. Lastly, there is a call for research that examines the cost-effectiveness and scalability of interventions, ensuring that successful strategies can be feasibly implemented at scale across diverse socio-economic contexts within the region. By addressing these implementation gaps, future research can better inform policy and public health strategies aimed at reducing the burden of type 2 diabetes in Asia-Pacific countries.

## Conclusion

In conclusion, lifestyle intervention may not be effective in reducing glycaemic outcomes but has an effect in reducing waist circumference according to the results of included studies in the meta-analysis in the Asia-Pacific region. Nevertheless, implementation outcomes suggested a potential cost-effectiveness in reducing waist circumference. This is encouraging, as central obesity is the strongest risk factor for cardiometabolic diseases in the East/Southeast region (a major part of the Asia Pacific Region). It is necessary to urgently design and implement not only effective but also feasible and sustainable lifestyle interventions and beyond to prevent T2DM in the Asia Pacific Region through all stakeholders.

The authors declare that they have no competing interests. This article does not contain any studies with human or animal subjects performed by any of the authors.

### Electronic Supplementary Material

Below is the link to the electronic supplementary material.

Supplementary Material 1

Supplementary Material 2

Supplementary Material 3

## Data Availability

No datasets were generated or analysed during the current study.
