# Supplementary material for: Lifestyle Intervention in Reducing Insulin Resistance and Preventing type 2 Diabetes in Asia Pacific Region: A Systematic Review and Meta-Analysis
Source: Curr Diab Rep. 2024 Jul 31;24(9):207–15. doi: 10.1007/s11892-024-01548-0 (PMC11303493; doi:10.1007/s11892-024-01548-0)
Supplement: Supplementary file 1 — Supplementary Material 1 [file 11892_2024_1548_MOESM1_ESM.docx]

**Search strategies used in database search**

The key search terms for searching Asia pacific region was referring to the list from Federal Aviation Administration, and use the key terms of “ Asia” OR “Southeast Asia” OR “South Aisa” OR “North Asia” OR “Oceania” OR “ Pacific Islands” OR “Afghanistan” OR “Bangladesh” OR “ Australia” OR “Bhutan” OR “Myanmar” OR “China” OR “Cambodia” OR “Brunei” OR “Polynesia” OR “Cook Islands” OR “Micronesia” OR “Fiji” OR “India” OR “Indonesia” OR “Japan” OR “Kiribati” OR “Laos” OR “Malaysia” OR “Maldives” OR “Marshall Islands” OR “Mongolia” OR “Nepal” OR “New Caledonia” OR “New Zealand” OR “Niue” OR “North Korea” OR “Pakistan” OR “Palau” OR “Philippines” OR “Singapore” OR “Solomon Islands” OR “South Korea” OR “Sri Lanka” OR “Taiwan” OR “Thailand” OR “Timor-Leste” OR “Tonga” OR “Tuvalu” OR “Vietanm”. For lifestyle interventions, the key terms included were “lifestyle modification” OR “diet* intervention” OR “physical activity” OR “exercise”. For diabetes, the key search terms used included “diabetes” OR “prediabetes” OR “impaired glucose tolerance” OR “diabetes risk” OR “insulin resistant”.

Search strategies used in different databases
