# Supplementary material for: Lifestyle Intervention in Reducing Insulin Resistance and Preventing type 2 Diabetes in Asia Pacific Region: A Systematic Review and Meta-Analysis
Source: Curr Diab Rep. 2024 Jul 31;24(9):207–15. doi: 10.1007/s11892-024-01548-0 (PMC11303493; doi:10.1007/s11892-024-01548-0)
Supplement: Supplementary file 2 — Supplementary Material 2 [file 11892_2024_1548_MOESM2_ESM.docx]

We found that 9 studies had good quality on risk assessment for treatment groups concealment (D2) whereas 16 studies were unclear on risk. The question on appropriateness on statistical analysis for all outcomes (D12) returned two unclear [1, 2] as compared to the rest (n=24). A risk assessment on follow-up completion, adequacy of its analysis, and description (D8) revealed one [3] on high risk given the reported association with attrition and intervention. 15 studies were ranked as unclear and 10 with low risk. Twenty-two studies followed appropriate trial design with no deviation from standard RCT (D13), whereas three [4-6] had unclear bias as no clear information was provided to justify using non-conventional RCT (e.g., cluster RCT) in the statistical methods and one study [2] had high risk as no justifications were done for the clustered RCT. A question on true randomization of participants’ assignment (D1) found that 8 studies had unclear bias, on the other hand, 18 had low risk. Fourteen studies had unclear findings on blinding of outcome accessors to treatment assignment (D6), two studies [7, 8] were at high risk and 10 studies were at low risk on assessment. Regarding the reliable way of measuring the outcome question (D11), most (n=19) were unclear and seven studies had good quality. All the studies (n=26) had good quality on risk assessment questions on outcome measurement for treatment groups (D10) but 31% of the studies (n=8) were unclear if the participants were analysed in the same group as randomized (D9) and have two [9, 10] were at high risk. Eleven studies were at high risk for participant blinding for treatment assignment question (D4), and only two studies had low risk and 13 unclear. A question regarding the blinding of those delivering treatment assignment (D5) revealed that only one study [3] had low risk ten studies had high risk and the remaining (n=15) were unclear. All the studies (n=23) had similar characteristics between groups at baseline (D3) except a few (n=3) [11-13] were ranked as unclear. All (n=24) studies had low risk on question that asked if the groups were treated identically other than the intervention of interest (D13), while two studies [2, 14] were unclear on their risk quality.

Reference

1. Moungngern Y, S.S., Teparak P, Sriwijitkamol A, *Effects of a Health Promotion Program Conducted by Nurses on Stabilization of HbA1C in Subjects at Risk for Diabetes: A Phase III Randomized Controlled Trial. .* J Med Assoc Thai 2018. **101:1343-8.**

2. Nguyen, S.N., et al., *Lifestyle Interventions Reduce the Risk of Type II Diabetes and Cardiovascular Diseases Development among Pre-diabetic Adults.* International Journal of Pharmaceutical Research and Allied Sciences, 2021. **10**(2): p. 94-102.

3. Pengpid, S., et al., *Effectiveness of a cluster-randomized controlled trial community-based lifestyle intervention program to control prehypertension and/or prediabetes in Thailand.* International Journal of Diabetes in Developing Countries, 2019. **39**(1): p. 123-131.

4. Aekplakorn, W., et al., *Evaluation of a Community-Based Diabetes Prevention Program in Thailand: A Cluster Randomized Controlled Trial.* J Prim Care Community Health, 2019. **10**: p. 2150132719847374.

5. Liu, Y., et al., *Short-term effects of lifestyle intervention in the reversion to normoglycemia in people with prediabetes.* Primary Care Diabetes, 2022. **16**(1): p. 168-172.

6. Sakane, N., et al., *Impact of telephone support programme using telemonitoring on stage of change towards healthy eating and active exercise in people with prediabetes.* Journal of Telemedicine and Telecare, 2021. **27**(5): p. 307-313.

7. Gong, Q., et al., *Efficacy of lifestyle intervention in adults with impaired glucose tolerance with and without impaired fasting plasma glucose: A post hoc analysis of Da Qing Diabetes Prevention Outcome Study.* Diabetes, Obesity and Metabolism, 2021. **23**(10): p. 2385-2394.

8. Raghuram, N., et al., *Effectiveness of a Yoga-Based Lifestyle Protocol (YLP) in Preventing Diabetes in a High-Risk Indian Cohort: A Multicenter Cluster-Randomized Controlled Trial (NMB-Trial).* Frontiers in Endocrinology, 2021. **12**.

9. Chen, X., et al., *The Effects of Mobile-App-Based Low-Carbohydrate Dietary Guidance on Postprandial Hyperglycemia in Adults with Prediabetes.* Diabetes Therapy, 2020. **11**(10): p. 2341-2355.

10. Guo, H., et al., *Effects of low-carbohydrate vs low-fat diets on weight loss and metabolic risk factors in obese/overweight individuals with impaired glucose regulation: A randomized controlled trial.* Asia Pacific Journal of Clinical Nutrition, 2022. **31**(3): p. 512-519.

11. Dai, X., et al., *Two-year-supervised resistance training prevented diabetes incidence in people with prediabetes: A randomised control trial.* Diabetes Metab Res Rev, 2019. **35**(5): p. e3143.

12. Teong, X.T., et al., *Intermittent fasting plus early time-restricted eating versus calorie restriction and standard care in adults at risk of type 2 diabetes: a randomized controlled trial.* Nature Medicine, 2023. **29**(4): p. 963-972.

13. Zhong, Q., et al., *The 18-month efficacy of an Intensive LifeStyle Modification Program (ILSM) to reduce type 2 diabetes risk among rural women: a cluster randomized controlled trial.* Globalization and Health, 2023. **19**(1): p. 6.

14. Kaur, H., N. Singla, and R. Jain, *Role of Nutrition Counseling and Lifestyle Modification in Managing Prediabetes.* Food Nutr Bull, 2021. **42**(4): p. 584-596.
