# Supplementary material for: Lifestyle Intervention in Reducing Insulin Resistance and Preventing type 2 Diabetes in Asia Pacific Region: A Systematic Review and Meta-Analysis
Source: Curr Diab Rep. 2024 Jul 31;24(9):207–15. doi: 10.1007/s11892-024-01548-0 (PMC11303493; doi:10.1007/s11892-024-01548-0)
Supplement: Supplementary file 3 — Supplementary Material 3 [file 11892_2024_1548_MOESM3_ESM.pdf]

MEDLINE

1

asia* pacific.mp. [mp=title, book title, abstract, original title, name of substance word, subject heading word, floating sub-heading word, keyword heading word, organism supplementary concept word, protocol supplementary concept word, rare disease supplementary concept word, unique identifier, synonyms, population supplementary concept word, anatomy supplementary concept word]

2

Asia, Eastern/ or Asia, Southern/ or Asia,Southeastern/ or Oceania/

3

1 or 2

4

exp Afghanistan/ or Afghanistan.mp. [mp=title, book title, abstract, original title, name of substance word, subject heading word, floating sub-heading word, keyword heading word, organism supplementary concept word, protocol supplementary concept word, rare disease supplementary concept word, unique identifier, synonyms, population supplementary concept word, anatomy supplementary concept word]

5

exp Australia/ or Australia.mp. [mp=title, book title, abstract, original title, name of substance word, subject heading word, floating sub-heading word, keyword heading word, organism supplementary concept word, protocol supplementary concept word, rare disease supplementary concept word, unique identifier, synonyms, population supplementary concept word, anatomy supplementary concept word]

6

exp Bangladesh/ or Bangladesh.mp. [mp=title, book title, abstract, original title, name of substance word, subject heading word, floating sub-heading word, keyword heading word, organism supplementary concept word, protocol supplementary concept word, rare disease supplementary concept word, unique identifier, synonyms, population supplementary concept word, anatomy supplementary concept word]

7

exp Bhutan/ or Bhutan.mp. [mp=title, book title, abstract, original title, name of substance word, subject heading word, floating sub-heading word, keyword heading word, organism supplementary concept word, protocol supplementary concept word, rare disease supplementary concept word, unique identifier, synonyms, population supplementary concept word, anatomy supplementary concept word]

8

exp Myanmar/ or Myanmar.mp. [mp=title, book title, abstract, original title, name of substance word, subject heading word, floating sub-heading word, keyword heading word, organism supplementary concept word, protocol supplementary concept word, rare disease supplementary concept word, unique identifier, synonyms, population supplementary concept word, anatomy supplementary concept word]

9

Burma.mp. [mp=title, book title, abstract, original title, name of substance word, subject heading word, floating sub-heading word, keyword heading word, organism supplementary concept word, protocol supplementary concept word, rare disease supplementary concept word, unique identifier, synonyms, population supplementary concept word, anatomy supplementary concept word]

10

exp Brunei/ or Brunei.mp. [mp=title, book title, abstract, original title, name of substance word, subject heading word, floating sub-heading word, keyword heading word, organism supplementary concept word, protocol supplementary concept word, rare disease supplementary concept word, unique identifier, synonyms, population supplementary concept word, anatomy supplementary concept word]

11

exp Cambodia/ or Cambodia.mp. [mp=title, book title, abstract, original title, name of substance word, subject heading word, floating sub-heading word, keyword heading word, organism supplementary concept word, protocol supplementary concept word, rare disease supplementary concept word, unique identifier, synonyms, population supplementary concept word, anatomy supplementary concept word]

12

exp China/ or China.mp. [mp=title, book title, abstract, original title, name of substance word, subject heading word, floating sub-heading word, keyword heading word, organism supplementary concept word, protocol supplementary concept word, rare disease supplementary concept word, unique identifier, synonyms, population supplementary concept word, anatomy supplementary concept word]

13

exp Polynesia/ or Polynesia.mp. [mp=title, book title, abstract, original title, name of substance word, subject heading word, floating sub-heading word, keyword heading word, organism supplementary concept word, protocol supplementary concept word, rare disease supplementary concept word, unique identifier, synonyms, population supplementary concept word, anatomy supplementary concept word]

14

Cook Islands.mp. [mp=title, book title, abstract, original title, name of substance word, subject heading word, floating sub-heading word, keyword heading word, organism supplementary concept word, protocol supplementary concept word, rare disease supplementary concept word, unique identifier, synonyms, population supplementary concept word, anatomy supplementary concept word]

15

exp Micronesia/ or Micronesia.mp. [mp=title, book title, abstract, original title, name of substance word, subject heading word, floating sub-heading word, keyword heading word, organism supplementary concept word, protocol supplementary concept word, rare disease supplementary concept word, unique identifier, synonyms, population supplementary concept word, anatomy supplementary concept word]

16

exp Fiji/ or Fiji.mp. [mp=title, book title, abstract, original title, name of substance word, subject heading word, floating sub-heading word, keyword heading word, organism supplementary concept word, protocol supplementary concept word, rare disease supplementary concept word, unique identifier, synonyms, population supplementary concept word, anatomy supplementary concept word]

17

exp India/ or India.mp. [mp=title, book title, abstract, original title, name of substance word, subject heading word, floating sub-heading word, keyword heading word, organism supplementary concept word, protocol supplementary concept word, rare disease supplementary concept word, unique identifier, synonyms, population supplementary concept word, anatomy supplementary concept word]

18

exp Indonesia/ or Indonesia.mp. [mp=title, book title, abstract, original title, name of substance word, subject heading word, floating sub-heading word, keyword heading word, organism supplementary concept word, protocol supplementary concept word, rare disease supplementary concept word, unique identifier, synonyms, population supplementary concept word, anatomy supplementary concept word]

19
exp Japan/ or Japan.mp. [mp=title, book title, abstract, original title, name of substance word, subject heading word, floating sub-heading word, keyword heading word, organism supplementary concept word, protocol supplementary concept word, rare disease supplementary concept word, unique identifier, synonyms, population supplementary concept word, anatomy supplementary concept word]
20
Kiribati.mp. [mp=title, book title, abstract, original title, name of substance word, subject heading word, floating sub-heading word, keyword heading word, organism supplementary concept word, protocol supplementary concept word, rare disease supplementary concept word, unique identifier, synonyms, population supplementary concept word, anatomy supplementary concept word]
21
exp Laos/ or Laos.mp. [mp=title, book title, abstract, original title, name of substance word, subject heading word, floating sub-heading word, keyword heading word, organism supplementary concept word, protocol supplementary concept word, rare disease supplementary concept word, unique identifier, synonyms, population supplementary concept word, anatomy supplementary concept word]
22
exp Malaysia/ or Malaysia.mp. [mp=title, book title, abstract, original title, name of substance word, subject heading word, floating sub-heading word, keyword heading word, organism supplementary concept word, protocol supplementary concept word, rare disease supplementary concept word, unique identifier, synonyms, population supplementary concept word, anatomy supplementary concept word]
23
exp Maldives/ or Maldives.mp. [mp=title, book title, abstract, original title, name of substance word, subject heading word, floating sub-heading word, keyword heading word, organism supplementary concept word, protocol supplementary concept word, rare disease supplementary concept word, unique identifier, synonyms, population supplementary concept word, anatomy supplementary concept word]
24
Marshall Islands.mp. [mp=title, book title, abstract, original title, name of substance word, subject heading word, floating sub-heading word, keyword heading word, organism supplementary concept word, protocol supplementary concept word, rare disease supplementary concept word, unique identifier, synonyms, population supplementary concept word, anatomy supplementary concept word]
25
exp Mongolia/ or Mongolia.mp. [mp=title, book title, abstract, original title, name of substance word, subject heading word, floating sub-heading word, keyword heading word, organism supplementary concept word, protocol supplementary concept word, rare disease supplementary concept word, unique identifier, synonyms, population supplementary concept word, anatomy supplementary concept word]
26
exp Nepal/ or Nepal.mp. [mp=title, book title, abstract, original title, name of substance word, subject heading word, floating sub-heading word, keyword heading word, organism supplementary concept word, protocol supplementary concept word, rare disease supplementary concept word, unique identifier, synonyms, population supplementary concept word, anatomy supplementary concept word]
27

exp New Caledonia/ or New Caledonia.mp. [mp=title, book title, abstract, original title, name of substance word, subject heading word, floating sub-heading word, keyword heading word, organism supplementary concept word, protocol supplementary concept word, rare disease supplementary concept word, unique identifier, synonyms, population supplementary concept word, anatomy supplementary concept word]

28

exp New Zealand/ or New Zealand.mp. [mp=title, book title, abstract, original title, name of substance word, subject heading word, floating sub-heading word, keyword heading word, organism supplementary concept word, protocol supplementary concept word, rare disease supplementary concept word, unique identifier, synonyms, population supplementary concept word, anatomy supplementary concept word]

29

exp Polynesia/ or Niue.mp. [mp=title, book title, abstract, original title, name of substance word, subject heading word, floating sub-heading word, keyword heading word, organism supplementary concept word, protocol supplementary concept word, rare disease supplementary concept word, unique identifier, synonyms, population supplementary concept word, anatomy supplementary concept word]

30

exp "Democratic People's Republic of Korea"/ or North Korea.mp. [mp=title, book title, abstract, original title, name of substance word, subject heading word, floating sub-heading word, keyword heading word, organism supplementary concept word, protocol supplementary concept word, rare disease supplementary concept word, unique identifier, synonyms, population supplementary concept word, anatomy supplementary concept word]

31

exp Pakistan/ or Pakistan.mp. [mp=title, book title, abstract, original title, name of substance word, subject heading word, floating sub-heading word, keyword heading word, organism supplementary concept word, protocol supplementary concept word, rare disease supplementary concept word, unique identifier, synonyms, population supplementary concept word, anatomy supplementary concept word]

32

exp Palau/ or Palau.mp. [mp=title, book title, abstract, original title, name of substance word, subject heading word, floating sub-heading word, keyword heading word, organism supplementary concept word, protocol supplementary concept word, rare disease supplementary concept word, unique identifier, synonyms, population supplementary concept word, anatomy supplementary concept word]

33

exp Papua New Guinea/ or Papua New Guinea.mp. [mp=title, book title, abstract, original title, name of substance word, subject heading word, floating sub-heading word, keyword heading word, organism supplementary concept word, protocol supplementary concept word, rare disease supplementary concept word, unique identifier, synonyms, population supplementary concept word, anatomy supplementary concept word]

34

exp Philippines/ or Philippines.mp. [mp=title, book title, abstract, original title, name of substance word, subject heading word, floating sub-heading word, keyword heading word, organism supplementary concept word, protocol supplementary concept word, rare disease supplementary concept word, unique identifier, synonyms, population supplementary concept word, anatomy supplementary concept word]

35

exp Singapore/ or Singapore.mp. [mp=title, book title, abstract, original title, name of substance word, subject heading word, floating sub-heading word, keyword heading word, organism supplementary concept word, protocol supplementary concept word, rare disease supplementary concept word, unique identifier, synonyms, population supplementary concept word, anatomy supplementary concept word]

36
exp Melanesia/ or Solomon Islands.mp. [mp=title, book title, abstract, original title, name of substance word, subject heading word, floating sub-heading word, keyword heading word, organism supplementary concept word, protocol supplementary concept word, rare disease supplementary concept word, unique identifier, synonyms, population supplementary concept word, anatomy supplementary concept word]
37
exp "Republic of Korea"/ or South Korea.mp. [mp=title, book title, abstract, original title, name of substance word, subject heading word, floating sub-heading word, keyword heading word, organism supplementary concept word, protocol supplementary concept word, rare disease supplementary concept word, unique identifier, synonyms, population supplementary concept word, anatomy supplementary concept word]
38
exp Sri Lanka/ or Sri Lanka.mp. [mp=title, book title, abstract, original title, name of substance word, subject heading word, floating sub-heading word, keyword heading word, organism supplementary concept word, protocol supplementary concept word, rare disease supplementary concept word, unique identifier, synonyms, population supplementary concept word, anatomy supplementary concept word]
39
exp Taiwan/ or Taiwan.mp. [mp=title, book title, abstract, original title, name of substance word, subject heading word, floating sub-heading word, keyword heading word, organism supplementary concept word, protocol supplementary concept word, rare disease supplementary concept word, unique identifier, synonyms, population supplementary concept word, anatomy supplementary concept word]
40
exp Thailand/ or Thailand.mp. [mp=title, book title, abstract, original title, name of substance word, subject heading word, floating sub-heading word, keyword heading word, organism supplementary concept word, protocol supplementary concept word, rare disease supplementary concept word, unique identifier, synonyms, population supplementary concept word, anatomy supplementary concept word]
41
exp Timor-Leste/ or Timor-Leste.mp. [mp=title, book title, abstract, original title, name of substance word, subject heading word, floating sub-heading word, keyword heading word, organism supplementary concept word, protocol supplementary concept word, rare disease supplementary concept word, unique identifier, synonyms, population supplementary concept word, anatomy supplementary concept word]
42
exp Tonga/ or Tonga.mp. [mp=title, book title, abstract, original title, name of substance word, subject heading word, floating sub-heading word, keyword heading word, organism supplementary concept word, protocol supplementary concept word, rare disease supplementary concept word, unique identifier, synonyms, population supplementary concept word, anatomy supplementary concept word]
43
exp Micronesia/ or Tuvalu.mp. [mp=title, book title, abstract, original title, name of substance word, subject heading word, floating sub-heading word, keyword heading word, organism supplementary concept word, protocol supplementary concept word, rare disease supplementary concept word, unique identifier, synonyms, population supplementary concept word, anatomy supplementary concept word]
44

exp Vanuatu/ or Vanuatu.mp. [mp=title, book title, abstract, original title, name of substance word, subject heading word, floating sub-heading word, keyword heading word, organism supplementary concept word, protocol supplementary concept word, rare disease supplementary concept word, unique identifier, synonyms, population supplementary concept word, anatomy supplementary concept word]

45

exp Vietnam/ or Vietnam.mp. [mp=title, book title, abstract, original title, name of substance word, subject heading word, floating sub-heading word, keyword heading word, organism supplementary concept word, protocol supplementary concept word, rare disease supplementary concept word, unique identifier, synonyms, population supplementary concept word, anatomy supplementary concept word]

46

4 or 5 or 6 or 7 or 8 or 9 or 10 or 11 or 12 or 13 or 14 or 15 or 16 or 17 or 18 or 19 or 20 or 21 or 22 or 23 or 24 or 25 or 26 or 27 or 28 or 29 or 30 or 31 or 32 or 33 or 34 or 35 or 36 or 37 or 38 or 39 or 40 or 41 or 42 or 43 or 44 or 45

47

3 or 46

48

exp Life Style/

49

exp Diet Therapy/

50

exp Exercise/

51

exp Smoking/

52

exp Diet/

53

exp Exercise Therapy/

54

exp Weight Loss/

55

48 or 49 or 50 or 51 or 52 or 53 or 54

56

("life style*" or "life style induced illness" or "lifestyle factor" or "health* behavio$r*" or "lifestyle modification").mp. [mp=title, book title, abstract, original title, name of substance word, subject heading word, floating sub-heading word, keyword heading word, organism supplementary concept word, protocol supplementary concept word, rare disease supplementary concept word, unique identifier, synonyms, population supplementary concept word, anatomy supplementary concept word]

57

("Diet* modification*" or "diet therap*" or "diet* restriction*" or "restrict* diet* therap*" or "diet* intervention*").mp. [mp=title, book title, abstract, original title, name of substance word, subject heading word, floating sub-heading word, keyword heading word, organism supplementary concept word,

protocol supplementary concept word, rare disease supplementary concept word, unique identifier, synonyms, population supplementary concept word, anatomy supplementary concept word]
58
("physical activit*" or "exercise intervention*" or "acute exercise*" or "aerobic exercise*" or "exercise training*" or "isometric exercise*").mp. [mp=title, book title, abstract, original title, name of substance word, subject heading word, floating sub-heading word, keyword heading word, organism supplementary concept word, protocol supplementary concept word, rare disease supplementary concept word, unique identifier, synonyms, population supplementary concept word, anatomy supplementary concept word]
59
('quit smoking' or 'quit alcohol' or 'smoking reduction' or 'alcohol reduction').mp. [mp=title, book title, abstract, original title, name of substance word, subject heading word, floating sub-heading word, keyword heading word, organism supplementary concept word, protocol supplementary concept word, rare disease supplementary concept word, unique identifier, synonyms, population supplementary concept word, anatomy supplementary concept word]
60
(weight adj3 (loss* or losing or lost or reduc* or chang* or control)).ti,ab.
61
((lifestyle* or life style* or behavior* or behaviour*) adj3 (intervention* or chang* or modif* or program* or therap* or treatment*)).ti,ab.
62
((diet* or nutrition* or calori*) adj2 (intervention* or chang* or modif* or program* or therap* or treatment* or restrict* or control* or counsel* or intak*)).ti,ab.
63
(physical* exerci* or exercise*).ti,ab.
64
56 or 61
65
57 or 62
66
58 or 63
67
59 or 60 or 64 or 65 or 66
68
55 or 67
69
exp Diabetes Mellitus/
70
Diabetes Mellitus/pc
71

Diabetes Mellitus, Type 2/pc
72
Prediabetic State/pc
73
Glucose Intolerance/pc
74
exp Insulin Resistance/
75
exp Blood Glucose/
76
exp Glucose Tolerance Test/
77
exp Body Mass Index/
78
exp Anthropometry/
79
exp Risk Factors/
80
69 or 70 or 71 or 72 or 73 or 74 or 75 or 76 or 77 or 78 or 79
81
(prediabet* or "pre-diabet*" or "type 2 diabet*" or "glucose intoleran*" or "diabetes mellitus" or "adult-onset diabetes mellitus" or "diabetes mellitus, type 2" or "diabetes, type 2" or "diabetes type ii").mp. [mp=title, book title, abstract, original title, name of substance word, subject heading word, floating sub-heading word, keyword heading word, organism supplementary concept word, protocol supplementary concept word, rare disease supplementary concept word, unique identifier, synonyms, population supplementary concept word, anatomy supplementary concept word]
82
((diabetes or type 2 diabetes or T2D$ or DM2 or NIDDM or noninsulin depend* or non-insulin depend*) adj4 (prevent* or reduc* or delay*)).ti,ab.
83
(risk adj2 (diabetes or type 2 diabetes or T2D$ or DM2 or NIDDM or noninsulin depend* or non-insulin depend*)).ti,ab.
84
(with adj (prediabetes or pre-diabetes or impaired glucose or impaired fasting or insulin resistance or glucose intolerance)).ti,ab.
85
((previous or prior or recent or after or history) adj2 (gestational diabetes or GDM)).ti,ab.
86
81 or 82 or 83 or 84 or 85
87
("insulin resistan*" or "insulin sensitive*" or OGTT or "Oral glucose tolerance test" or "tolerance test" or "2-hour glucose").mp. [mp=title, book title, abstract, original title, name of substance word, subject heading word, floating sub-heading word, keyword heading word, organism supplementary

concept word, protocol supplementary concept word, rare disease supplementary concept word, unique identifier, synonyms, population supplementary concept word, anatomy supplementary concept word]

88

("fasting blood glucose" or "blood glucose" or "blood sugar").mp. [mp=title, book title, abstract, original title, name of substance word, subject heading word, floating sub-heading word, keyword heading word, organism supplementary concept word, protocol supplementary concept word, rare disease supplementary concept word, unique identifier, synonyms, population supplementary concept word, anatomy supplementary concept word]

89

"postprandial glucose".mp. [mp=title, book title, abstract, original title, name of substance word, subject heading word, floating sub-heading word, keyword heading word, organism supplementary concept word, protocol supplementary concept word, rare disease supplementary concept word, unique identifier, synonyms, population supplementary concept word, anatomy supplementary concept word]

90

(BMI or "body mass index" or "quetelet* index").mp. [mp=title, book title, abstract, original title, name of substance word, subject heading word, floating sub-heading word, keyword heading word, organism supplementary concept word, protocol supplementary concept word, rare disease supplementary concept word, unique identifier, synonyms, population supplementary concept word, anatomy supplementary concept word]

91

(anthropomet* or "body composition" or "body weight* and measure*").mp. [mp=title, book title, abstract, original title, name of substance word, subject heading word, floating sub-heading word, keyword heading word, organism supplementary concept word, protocol supplementary concept word, rare disease supplementary concept word, unique identifier, synonyms, population supplementary concept word, anatomy supplementary concept word]

92

("risk factor*" or "risk score*" or "diabetes risk score*" or "diabetes risk assessment tool").mp. [mp=title, book title, abstract, original title, name of substance word, subject heading word, floating sub-heading word, keyword heading word, organism supplementary concept word, protocol supplementary concept word, rare disease supplementary concept word, unique identifier, synonyms, population supplementary concept word, anatomy supplementary concept word]

93

86 or 87 or 88 or 89 or 90 or 91 or 92

94

80 or 93

95

47 and 68 and 94

96

limit 95 to last 10 years

EMBASE

1
diabetes.mp. or exp diabetes mellitus/
2
prediabetes.mp. or impaired glucose tolerance/
3
diabetes risk.mp.
4
diet* intervention.mp. or diet therapy/
5
diet/ or diet therapy/ or diet.mp. or diet restriction/
6
physical activity.mp. or exp physical activity/
7
exp exercise/ or exercise.mp.
8
lifestyle modification.mp. or exp lifestyle/ or exp lifestyle modification/
9
life style.mp. or lifestyle/
10
weight loss.mp. or body weight loss/
11
insulin resistance.mp. or insulin resistance/
12
risk factor/ or diabetes risk score.mp. or diabetes risk score/
13
asia.mp. or Asia/ or Southeast Asia/ or South Asia/ or northern Asia/
14
Oceania.mp. or Pacific islands/
15
Afghanistan.mp. or exp Afghanistan/
16
Bangladesh.mp. or exp Bangladesh/
17
exp Australia/ or Australia.mp.
18

Bhutan.mp. or exp Bhutan/
19
Myanmar.mp. or exp Myanmar/
20
Burma.mp. or exp Myanmar/
21
china.mp. or exp China/
22
cambodia.mp. or exp Cambodia/
23
brunei.mp. or exp Brunei Darussalam/
24
exp Polynesia/ or Polynesia.mp.
25
cook islands.mp. or exp Cook Islands/
26
Micronesia.mp. or exp "Federated States of Micronesia"/
27
Fiji.mp. or exp Fiji/
28
exp India/ or India.mp.
29
Indonesia.mp. or exp Indonesia/
30
exp Japan/ or Japan.mp.
31
Kiribati.mp. or exp Kiribati/
32
Laos.mp. or exp Laos/
33
malaysia.mp. or exp Malaysia/
34
maldives.mp. or exp Maldives/
35
exp Marshall Islands/ or marshall islands.mp.
36
exp Mongolia/ or Mongolia.mp.

37
nepal.mp. or exp Nepal/
38
New Caledonia.mp. or exp New Caledonia/
39
New zealand.mp. or exp New Zealand/
40
exp Niue/
41
North Korea.mp. or exp North Korea/
42
Pakistan.mp. or exp Pakistan/
43
Palau.mp. or exp Palau/
44
Papua new guinea.mp. or exp Papua New Guinea/
45
philippines.mp. or exp Philippines/
46
singapore.mp. or exp Singapore/
47
exp Solomon Islands/ or solomon islands.mp.
48
South Korea.mp. or exp South Korea/
49
Sri Lanka.mp. or exp Sri Lanka/
50
exp Taiwan/ or Taiwan.mp.
51
exp Thailand/ or Thailand.mp.
52
Timor-Leste.mp. or exp Timor-Leste/
53
Tonga.mp. or exp Tonga/
54
Tuvalu.mp. or exp Tuvalu/
55

Vanuatu.mp. or exp Vanuatu/
56
vietnam.mp. or exp Viet Nam/
57
13 or 14 or 15 or 16 or 17 or 18 or 19 or 20 or 21 or 22 or 23 or 24 or 25 or 26 or 27 or 28 or 29 or 30 or 31 or 32 or 33 or 34 or 35 or 36 or 37 or 38 or 39 or 40 or 41 or 42 or 43 or 44 or 45 or 46 or 47 or 48 or 49 or 50 or 51 or 52 or 53 or 54 or 55 or 56
58
1 or 2 or 3 or 11 or 12
59
4 or 5 or 6 or 7 or 8 or 9 or 10
60
57 and 58 and 59
61
limit 60 to (full text and english language and yr="2013 - 2023")

CINAHL

Search ID#	Search Terms	Search Options	[illegible]
S12	S1 AND S8 AND S9	**Limiters** - Full Text; Published Date: 20180101-20231231<br>**Expanders** - Apply equivalent subjects<br>**Search modes** - Boolean/Phrase	[illegible]
S11	S1 AND S8 AND S9	**Limiters** - Full Text<br>**Expanders** - Apply equivalent subjects<br>**Search modes** - Boolean/Phrase	[illegible]
S10	S1 AND S8 AND S9	**Expanders** - Apply equivalent subjects<br>**Search modes** - Boolean/Phrase	[illegible]
S9	S5 OR S6 OR S7	**Expanders** - Apply equivalent subjects<br>**Search modes** - Boolean/Phrase	[illegible]
S8	S2 OR S3	**Expanders** - Apply equivalent subjects<br>**Search modes** - Boolean/Phrase	[illegible]

S7	thailand OR timor OR tonga OR tuvalu OR vanuatu OR vietnam	**Expanders** - Apply equivalent subjects<br>**Search modes** - Boolean/Phrase	
S6	New Caledonia OR new zealand OR niue OR north korea OR palau OR papua new guinea OR philippines OR singapore OR solomon OR south korea OR sri lanka OR taiwan	**Expanders** - Apply equivalent subjects<br>**Search modes** - Boolean/Phrase	
S5	Polynesia OR cook islands OR Fiji OR Micronesia OR indonesia OR kiribati OR laos OR malyasia OR maldives OR marshall islands OR mongolia OR nepal	**Expanders** - Apply equivalent subjects<br>**Search modes** - Boolean/Phrase	
S4	MW asia pacific OR Asia OR (south asian or indian or pakistani or bangladeshi) OR northeast asia OR southeast asia OR oceania OR afghanistan OR australia OR Bhutan OR Brunei OR Cambodia OR China	**Expanders** - Apply equivalent subjects<br>**Search modes** - Boolean/Phrase	
S3	MW weight loss or weight reduction or lose weight or weight management	**Expanders** - Apply equivalent subjects<br>**Search modes** - Boolean/Phrase	
S2	MH (lifestyle changes or lifestyle modification or lifestyle choices) OR MH (smoking cessation or smoking cessation interventions or quit smoking or stop smoking) OR MH tobacco use cessation OR (lifestyle intervention or lifestyle change or diet or exercise) OR MH dietary intervention OR (dietary modifications or diet modifications or diet or healthy diet) OR physical activity OR physical therapy OR alcohol reduction	**Expanders** - Apply equivalent subjects<br>**Search modes** - Boolean/Phrase	
S1	MH (diabetes type 2 or diabetes mellitus type 2 or diabetes 2) OR MH (prediabetes or impaired glucose tolerance or impaired fasting glucose) OR MH glucose intolerance OR high risk diabetes OR MH insulin resistance OR (fasting glucose or fasting blood sugar or fasting serum or fasting ins		

Cochrane Centre for registered trials

ID	Search
#1	type 2 diabetes mellitus with Publication Year from 2013 to 2023, in Trials
#2	prediabetes with Publication Year from 2013 to 2023, in Trials
#3	insulin resistance with Publication Year from 2013 to 2023, in Trials
#4	glucose intolerance with Publication Year from 2013 to 2023, in Trials
#5	#1 or #2 or #3 or #4 with Publication Year from 2013 to 2023, in Trials
#6	lifestyle intervention with Publication Year from 2013 to 2023, in Trials
#7	lifestyle modification with Publication Year from 2013 to 2023, in Trials
#8	dietary intervention with Publication Year from 2013 to 2023, in Trials
#9	"nutrition" intervention or "nutrition modif NEXT" or "nutrition program" with Publication Year from 2013 to 2023, in Trials
#10	exercise with Publication Year from 2013 to 2023, in Trials
#11	physical activity with Publication Year from 2013 to 2023, in Trials
#12	weight loss with Publication Year from 2013 to 2023, in Trials
#13	#6 or #7 or #8 or #9 or #10 or #11 or #12
#14	Asia or South Asia or Southeast Asia or Asia Pacific or Oceania with Publication Year from 2013 to 2023, in Trials
#15	Afghanistan or Bangladesh or Australia or Bhutan or Myanmar or Burma or China or Taiwan or Cambodia or Brunei or Polynesia or Cook Islands or Micronesia or Fiji or India or Indonesia or Japan or Kiribati or Laos or Malaysia or Maldives or Marshall Islands or Mongolia or Nepal or New Caledonia or New Zealand or Niue or North Korea or Pakistan or Palau or Papua or Philippines or Singapore or Solomon Islands or South Korea or Sri Lanka or Thailand or Timor Leste or Tonga or Tuvalu or Vanuatu or Vietnam with Publication Year from 2013 to 2023, in Trials
#16	#14 or #15 with Publication Year from 2013 to present, in Trials

#17 #5 and #13 and #16 with Publication Year from 2013 to 2023, in Trials
